# SARS-CoV-2 mRNA Vaccines: Immunological Mechanism and Beyond

**DOI:** 10.3390/vaccines9020147

**Published:** 2021-02-12

**Authors:** Emily Bettini, Michela Locci

**Affiliations:** Department of Microbiology, Perelman School of Medicine, University of Pennsylvania, Philadelphia, PA 19104, USA; ebettini@pennmedicine.upenn.edu

**Keywords:** SARS-CoV-2, coronavirus, mRNA vaccines, adaptive immunity, antibodies, germinal centers, long-lived plasma cells, memory B cells, T follicular helper cells, Th1 cells

## Abstract

To successfully protect against pathogen infection, a vaccine must elicit efficient adaptive immunity, including B and T cell responses. While B cell responses are key, as they can mediate antibody-dependent protection, T cells can modulate B cell activity and directly contribute to the elimination of pathogen-infected cells. In the unprecedented race to develop an effective vaccine for severe acute respiratory syndrome coronavirus 2 (SARS-CoV-2), the causative agent of the respiratory disease coronavirus disease 2019 (COVID-19), messenger RNA (mRNA) vaccines have emerged as front runners thanks to their capacity for rapid development and ability to drive potent adaptive immune responses. In this review article, we provide an overview of the results from pre-clinical studies in animal models as well as clinical studies in humans that assessed the efficacy of SARS-CoV-2 mRNA vaccines, with a primary focus on adaptive immune responses post vaccination.

## 1. Introduction

In December of 2019, the betacoronavirus severe acute respiratory syndrome coronavirus 2 (SARS-CoV-2), was identified as the causative agent of the debilitating coronavirus disease 2019 (COVID-19). At the time this review was written (December 2020), this disease had led to over 80 million infections and 1.8 million deaths globally [1], highlighting the crucial need to develop a safe and effective vaccine. Thus far, there are 61 SARS-CoV-2 vaccine candidates already under clinical evaluation and 172 in preclinical development [2]. In this unprecedented effort to generate one or more successful vaccines for SARS-CoV-2, many different vaccine platforms have been deployed. Several of the candidates currently under investigation are based on traditional vaccine approaches, such as inactivated/live-attenuated virus or recombinant proteins (detailed in [3]). Others exploit novel and promising platforms, like the nucleic acid-based messenger RNA (mRNA) vaccines that deliver the genetic information to produce the antigen, rather than the antigen itself. While conventional vaccine approaches have been safely and ubiquitously used for many decades, the unknowns of mRNA vaccination are many, seeing as this is a first-in-class licensure for mRNA vaccines. After briefly discussing some crucial aspects of design strategy for mRNA vaccines, this review will highlight our current knowledge on the adaptive immune responses to SARS-CoV-2 mRNA vaccines based on animal studies from many different groups, as well as clinical data available from the SARS-CoV-2 mRNA vaccines mRNA-1273, co-developed by Moderna (Cambridge, MA, USA) and the National Institutes of Allergy and Infectious Diseases Vaccine Research Center, along with BNT162b1 and BNT162b2, from BioNTech (Mainz, Germany)/Pfizer (New York City, NY, USA). Furthermore, the major strengths and challenges associated with the usage of mRNA vaccines will also be outlined here. We must also note that, although there are promising self-replicating RNA candidates currently in clinical development, this review will only focus on SARS-CoV-2 mRNA vaccine candidates.

## 2. Design Strategies for SARS-CoV-2 mRNA Vaccines

The conceptualization of mRNA vaccines might seem quite simple at first, due to the straightforward *modus operandi* of mRNA vaccines. Upon delivery of an mRNA vaccine encoding a target antigen, cells will take up the mRNA and translate it into protein in situ. The individual’s immune system will then mount a robust adaptive immune response against the target protein. Nevertheless, the actual design process of mRNA vaccines requires important considerations of mRNA modifications to reduce reactogenicity and optimize protein expression; proper selection of the target antigen; and optimal formulation allowing for an efficient delivery [4].

### 2.1. Modifications to mRNA

The first aspect to consider in the development of mRNA vaccines is that unmodified mRNA itself is not ideal for use in vaccine development [4]. In fact, mRNA is both extremely labile and rapidly degraded in unfavorable environments. Moreover, it is highly immunogenic and able to activate a variety of pathogen-associated molecular pattern sensors. In an effort to improve half-life as well as translatability and safety, Karikó et al. tested a variety of naturally occurring modifications to nucleosides in mRNA molecules, including pseudouridine, 5-methylcytidine, N6-methyladenosine, 5-methyluridine, and 2-thiouridine [5]. Of these variants, they found that the incorporation of N1-methyl-pseudouridine (m1Ψ) in place of uridine led to a 10-fold increase in translation over unmodified mRNA. Furthermore, they were able to show that mRNA molecules possessing this modification did not trigger pathogen-associated molecular pattern sensing mechanisms such as toll-like receptors (TLRs) or retinoic acid-inducible gene I (RIG-I) [4,5]. This is crucial to avoid excessive inflammation, which could result in undesired vaccine side-effects. For these reasons, many candidates, including the two recently licensed mRNA vaccines mRNA-1273 and BNT162b2, adopted this m1Ψ mRNA modification in their vaccine design [6,7,8,9,10,11,12,13,14,15,16]. It is important to note here that, although there are other potential modifications that vaccine manufacturers can apply to mRNA molecules, m1Ψ is the most ubiquitously used modification, and it will be the only mRNA modification discussed in this review.

### 2.2. Antigen Selection

In selecting the antigen for an mRNA vaccine, it is essential to choose a target that is both immunogenic and capable of eliciting a protective immune response. Of the multiple epitopes on SARS-CoV-2, the spike (S) glycoprotein is the target commonly selected for COVID-19 vaccine development [3], since it is the major SARS-CoV-2 surface protein and mediates viral entry by binding to the angiotensin-converting enzyme 2 (ACE2) receptor in host cells [17,18,19]. SARS-CoV-2 S is a class I viral fusion glycoprotein, consisting of a receptor binding subunit (S1) and a fusion subunit (S2) that are joined by a furin cleavage site unique to this coronavirus (Figure S1) [18,20]. S is cleaved post-translationally at this furin site. However, the S1 and S2 subunits stay associated until S is bound to the ACE2 receptor via the receptor binding domain (RBD), leading to irreversible conformational changes and membrane fusion. Information gleaned from previous work with similar fusion glycoproteins has shown how important it is to use prefusion stabilized proteins that preserve neutralization-sensitive epitopes for the development of effective vaccines [21,22]. To stabilize the S protein, a few different strategies have been adopted (Figure S1). A mutation where amino acids 986 and 987 are replaced with prolines (S-2P), stabilizing the transmembrane-anchored S glycoprotein in the prefusion conformation but still allowing for cleavage of the S1 and S2 subunits [17,21], is the approach used in the licensed vaccines mRNA-1273 and BNT162b2 [6,7,8,9,10,11,12,15,16]. Another approach consisted of designing an mRNA construct where the full-length S protein lacks the furin cleavage site (Δfurin) and cannot be cleaved after translation [23,24].

As an alternative to the full-length S protein, RBD could exclusively be targeted as an antigen by mRNA vaccines (Figure S1), as several studies have shown that the many neutralizing epitopes are included within RBD [25,26,27,28,29]. One of the clinical candidates originally developed by BioNTech/Pfizer and tested in early clinical studies, BNT162b1 [13,14,15], encoded a secreted trimerized RBD. The choice of either full-length S or RBD might have different advantages. For instance, while RBD is enriched for epitopes important for SARS-CoV-2 neutralization, the full-length S is a notably bigger protein and contains additional epitopes that might be important to elicit broader adaptive immune responses.

### 2.3. Delivery of mRNA

Finally, although naked mRNA can be directly injected for immunization, this method of delivery is rather inefficient. Indeed, mRNA molecules must be able to penetrate a cell’s lipid membrane in order to reach the machinery required to translate the transcripts into proteins. Thus, delivery methods facilitating the cytosolic localization of mRNA vaccines are important for achieving efficient protein translation. In early studies, standard laboratory lipid encapsulation methods, such as lipofectamine [5], worked well for in vitro applications. These methods, however, had limited efficacy when tested in vivo and were also found to be highly cytotoxic [4]. The advent of lipid nanoparticle (LNP) encapsulation was a turning point in the development of mRNA vaccines, as LNPs are able to efficiently deliver mRNA in vivo [30,31]. When injected intramuscularly, mRNA-LNPs can be internalized and quickly translated by antigen-presenting cells at both the injection site and in draining lymph nodes, thus promoting the initiation of adaptive immune responses [32]. Additionally, LNPs can protect mRNA from degradation by nucleases. Although the precise composition of the LNPs used by many vaccine developers is proprietary information, it is known that LNPs contain a combination of ionizable cationic lipids, cholesterol, phospholipid and PEGs that self-assemble into ~100 nm nanoparticles encapsulating the mRNA [33,34,35]. Of the vaccine candidates in clinical trials, LNPs are the standard method being used to introduce mRNA vaccines to participants. Additional delivery methods that are not used by any SARS-CoV-2 preclinical or clinical candidates have been thoroughly reviewed elsewhere [4].

## 3. Immune Responses Elicited by SARS-CoV-2 mRNA Vaccines: Lessons from Animal Studies

**Figure 1:**
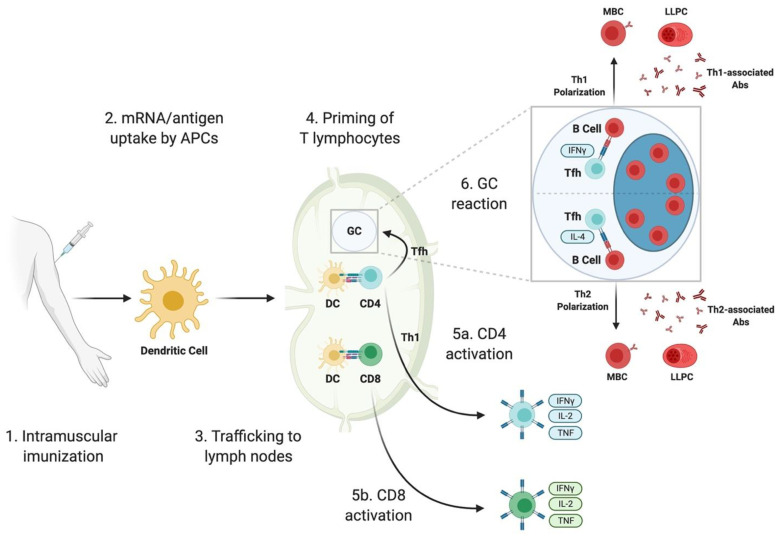
Immune responses elicited by SARS-CoV-2 mRNA vaccines. SARS-CoV-2 mRNA vaccines are administered intramuscularly (1). Either mRNA-LNPs or locally produced antigen are taken up by antigen-presenting cells (APCs) (2), such as dendritic cells (DCs). These APCs then traffic to the lymph nodes (3) where they are able to prime CD4 and CD8 T lymphocytes (4). The events in (2)–(4) are reviewed in detail in [36]. The priming of CD8 T cells can induce the formation of cytotoxic T lymphocytes (5b) which are capable of directly killing infected cells. Antigen-primed CD4 T cells can differentiate into Th1 cells (5a) or T follicular helper (Tfh) cells. Tfh cells help to initiate a germinal center (GC) reaction (6). GC reactions induced by vaccination will result in the formation of affinity-matured memory B cells (MBCs) and the antibody-secreting long-lived plasma cells (LLPCs). Tfh cells can be skewed towards either a Th1 or Th2 phenotype, which will affect the class switching of antibodies (Abs) produced by LLPCs to either Th1- or Th2-associated Abs (6).

A schematic representation of the immune responses elicited by SARS-CoV-2 mRNA vaccines, built on the data from animal studies discussed in this section, is depicted in Figure 1. While mRNA vaccine uptake/biodistribution and the innate immune response to mRNA vaccines are critical for the initiation of adaptive immunity, these processes are thoroughly reviewed elsewhere [36] and will not be covered in this article due to a lack of available data generated in the context of SARS-CoV-2 mRNA vaccines.

### 3.1. B Cells and Antibody Responses

Most licensed vaccines confer protection from viral infection by eliciting protective antibody (Ab) responses that last over time [37]. Affinity-matured, long-lasting Abs against viruses are generated in microanatomical sites of secondary lymphoid organs named germinal centers (GCs) [38]. In GCs, antigen-activated B cells undergo random mutations and diversify their immunoglobulin genes to generate Abs with high affinity for the pathogen. Next, only the B cells that have acquired higher affinity are positively selected and rescued from apoptosis. This sophisticated Darwinian process ultimately leads to the formation of high-affinity long-lived plasma cells (LLPCs) and memory B cells (MBCs). LLPCs secrete Abs, some of which are neutralizing Abs (nAbs) and can potentially mediate sterilizing immunity [39]. Importantly, LLPCs can survive for years, or even several decades in some cases, continuously secreting Abs without the need for further antigen stimulation [40]. Conversely, MBCs become activated in the event of a subsequent pathogen exposure and give rise to a new burst of high-affinity Ab-secreting cells [39]. Hence, both LLPC and MBCs are desirable cell types to induce by vaccination.

#### 3.1.1. Induction of SARS-CoV-2 Binding and Neutralizing Abs

Studies of natural infection in humans have shown that individuals infected with SARS-CoV-2 can produce potent neutralizing Abs (targeting the SARS-CoV-2 S protein) that might inhibit infection by SARS-CoV-2 in vitro and/or in vivo [29,41]. Based on this knowledge, pre-clinical evaluations of SARS-CoV-2 mRNA vaccines have focused on the ability of these vaccines to elicit robust SARS-CoV-2-binding and neutralizing Ab responses in mice, as an initial step in assessing vaccine efficacy. Work done by our group has shown that a single 30μg dose of an mRNA-LNP vaccine, encoding either the full-length Δfurin S or the RBD of SARS-CoV-2 (Figure S1), was able to promote high SARS-CoV-2-binding immunoglobulin G (IgG) titers in mice as early as two weeks post-immunization [23,24]. Despite a high vaccine dose being utilized in our work [23,24], studies of the clinical candidates mRNA-1273 (Moderna), BNT162b2 (BioNTech/Pfizer) and CVnCoV (CureVac (Frankfurt, Germany)), all encoding a full-length, pre-fusion stabilized S protein (Figure S1) [8,12,42] and ARCoV [43], encoding the RBD, reported that the production of SARS-CoV-2-specific Abs was driven by a significantly lower dose (ranging from 0.2 to 10 μg) of these vaccine candidates in mice. Importantly, various groups found that the Ab responses induced by these SARS-CoV-2 mRNA vaccines were able to neutralize the virus in vitro, as measured by both pseudovirus- and SARS-CoV-2-based neutralization assays [8,12,23,24,42,43,44,45], and that nAb levels were sustained for two months, or more, post-immunization [23,24,44,45]. Analyses of the Ab responses elicited by different vaccine doses indicated that mRNA vaccines induced a dose-dependent SARS-CoV-2-specific Ab response upon priming that was enhanced by a booster immunization [8,24,42,43]. While a single immunization with a high vaccine dose (30 μg) appeared sufficient to promote an elevated humoral response in mice [23,24], a booster immunization was necessary for nAb generation at the lower vaccine doses (1 μg or 2 μg) [8,42,43]. Altogether, the data from these different mouse studies suggest that either high doses of mRNA or a booster immunization might be required to reach detectable nAb levels after immunization with SARS-CoV-2 mRNA vaccines. Interestingly, the antigen choice seems to have an impact on a vaccine’s capacity to drive Ab responses, as indicated by a study wherein a SARS-CoV-2 S1 mRNA vaccine (Figure S1) was not as effective at inducing SARS-CoV-2-binding Abs and nAbs as the RBD mRNA vaccines [45]. This is different from the potent humoral responses observed by several other groups utilizing mRNA vaccines encoding either the S Δfurin [23,24] or the S-2P constructs [8,12,42], suggesting that a full-length S, where the S1 and S2 subunits are both present, might be a better immunogen than the simple S1 subunit.

Beyond mice, studies in non-human primates (NHPs) are instrumental in determining the efficacy of vaccine candidates for SARS-CoV-2, as the wild-type virus cannot efficiently replicate in common laboratory mouse strains, due to the lack of appropriate receptors to initiate viral infection [46]. In NHPs, the clinical candidates mRNA-1273 (10 or 100 μg) and BNT162b2 (30 or 100 μg) both demonstrated a robust, dose-dependent capacity to elicit SARS-CoV-2 specific Abs after two immunizations [7,12]. A similar trend was also observed for the in vitro neutralizing capacity of these Abs [7,12], with nAb values elicited in NHPs by BNT162b2 that were even higher than those from SARS-CoV-2 convalescent human sera [12]. The candidate CVnCoV was tested at much lower dosages (0.5 or 8 μg), but only the higher dosage elicited significant levels of S- and RBD-binding IgG titers after a single immunization, which increased over time [47]. In all cases, the nAb responses elicited by these candidates were combined with in vivo protection against SARS-CoV-2 challenge after the booster immunization [7,12,47]. Although viral replication was detected in the upper respiratory tract of the infected animals that received any of these vaccines, it was transient and only measurable for a few days. Importantly, the lower respiratory tract of immunized NHPs was fully protected from viral replication, as indicated by the absence of measurable SARS-CoV-2 subgenomic RNA in bronchoalveolar lavage fluid at any time point [7]. These studies also demonstrated that the higher vaccine doses conferred greater protection of the upper respiratory tract, while both dosages were equally effective in protecting the lower respiratory tract [7,12].

For coronavirus vaccine development, there are some noteworthy concerns when it comes to the quality of Ab production. One concern is that SARS-CoV-2 vaccines might favor the development of Ab-dependent enhancement, a phenomenon that could occur when vaccine-induced Abs fail to effectively neutralize viruses because of insufficient concentration/affinity, or the wrong specificity [48]. While no evidence has shown that Ab-dependent enhancement can occur in SARS-CoV-2 infection, the robust levels of SARS-CoV-2 binding Abs and nAbs driven by all SARS-CoV-2 mRNA vaccines discussed above, combined with the in vivo protection data in NHPs, seem favorable in order to avoid any potential for Ab-dependent enhancement.

#### 3.1.2. Germinal Center-Derived B Cell Response

GC reactions are fundamental for the generation of high-quality B cell responses that can confer protection over an extended time. Data from our group demonstrated that a single 30 μg dose of a SARS-CoV-2 mRNA vaccine, encoding either the full-length Δfurin S protein or the RBD, was able to promote robust formation of primary GCs 7 days post-immunization in mice [24]. This was opposed to a minimal GC induction in response to the immunization with the more traditional recombinant RBD (rRBD) protein vaccine, formulated with the MF59-like adjuvant AddaVax (rRBD-AddaVax). In this study, GCs were evaluated by both flow cytometry and microscopy. Similarly, GC induction by SARS-CoV-2 mRNA vaccines in mice was also described by two additional studies: Vogel et al. showed a significant increase in GC B cells in both the spleen and the draining lymph nodes 12 days after immunization with a single 5 μg dose of BNT162b2 [12], whereas Tai et al. [45] observed the presence of GCs 10 days after a booster immunization with a 30 μg dose of SARS-CoV-2 mRNA vaccines.

In our study, the GC B cells elicited by a single dose of mRNA vaccines were SARS-CoV-2-specific and peaked around day 7, then progressively waned over time and were mostly resolved by day 28 [24]. Such a GC kinetic in response to mRNA vaccines can be considered fast, as primary GC responses to protein antigens in the adjuvant usually peak between days 10 and 14 post-immunization [49]. Interestingly, although there was a deep quantitative reduction in GCs at week 4 post-immunization, the elevated frequency of SARS-CoV-2-specific GC B cells at this time point indicated that some residual low-level GC activity was still present in response to mRNA vaccines. This residual GC activity could contribute to the elevated SARS-CoV-2-specific IgG titers and nAbs found 60 days post-immunization [24]. Additionally, a booster immunization resulted in a second wave of SARS-CoV-2-specifc GC B cell formation measured 10 days post-boost.

As discussed above, the most desirable B cell populations to generate by vaccination are affinity matured LLPCs and MBCs, cell types canonically generated through GC reactions. In two recent studies from our group, we examined GC-derived B cell responses generated in mice by a single immunization with SARS-CoV-2 mRNA vaccines, starting from the analysis of LLPCs [23,24]. Specifically, we observed a high level of SARS-CoV-2-specific Ab-secreting cells in the bone marrow of mice immunized 9 weeks earlier with SARS-CoV-2 mRNA vaccines. Because of the location and time point of analysis, these data were indicative of the successful generation of LLPCs following the GC reactions. In these studies, SARS-CoV-2-specific MBCs were also evaluated post-immunization with mRNA vaccines. Consistent with the detection of memory precursors (CCR6-expressing cells in GC light zones) during the early phase of immune responses [24], we further demonstrated that there was a measurable pool of antigen-specific, class-switched MBC 60 days post-vaccination [23,24].

The data emerging from Lederer et al. further illustrated that vaccination with SARS-CoV-2 mRNA vaccines resulted in more efficient nAb production in comparison to rRBD-AddaVax, as measured by both microneutralization and pseudoneutralization assays [24]. Importantly, we observed a strong correlation between the magnitude of GC responses and the levels of nAbs induced by SARS-CoV-2 vaccination, suggesting that the formation of GCs could be necessary for a neutralizing response to SARS-CoV-2. It is worth noting that our finding linking GC reactions to efficient nAb responses upon immunization is apparently discordant with data from SARS-CoV-2 natural infection in humans. Several studies have indeed described that near-germline nAbs endowed with potent in vitro neutralization activity are elicited during SARS-CoV-2 infection, and are characterized by low-grade mutations [27,50,51,52]. These data are indicative of a limited GC process involved in the generation of such nAbs. Nevertheless, it is important to remember that GCs do not only harbor the affinity maturation process, but are also important for generating LLPCs that live for extended times and mediate durable Ab production. It is therefore possible that the plasma cells producing the near-germline nAbs might have a limited lifespan. While the majority of the studies on the longevity of Ab responses to SARS-CoV-2 upon infection have shown that SARS-CoV-2-binding IgG are only moderately reduced at 5–8 months post-infection [53,54], there are some conflicting results regarding the longevity of nAbs. Some studies indeed suggested that COVID-19 might generate slowly waning nAbs [53,54,55]. Conversely, others reported a steeper decline in nAbs over time [56,57], which could indicate that natural SARS-CoV-2 infection might sometimes elicit nAb responses of limited durability. Additionally, in a postmortem evaluation of lymph nodes from severe COVID-19 donors, a profound decrease in/lack of GC formation was recently described by Pillai and colleagues [58]. This study suggests that natural SARS-CoV-2 infection might not efficiently generate GC-derived long-lasting humoral immunity, at least in the most severe COVID-19 cases. While future studies will be important to assess the actual longevity of Ab responses elicited by SARS-CoV-2 natural infection, with special emphasis on nAb responses, it appears highly desirable that a SARS-CoV-2 mRNA vaccine might induce robust GC responses in order to potentially give rise to long-lasting serological memory and MBCs.

### 3.2. T Cell Responses

While vaccine-induced protection largely relies on Ab responses [39], effective vaccine candidates for SARS-CoV-2 could greatly benefit from the induction of T cell responses, for multiple reasons. Firstly because, among CD4 T cells, T follicular helper (Tfh) cells are crucial regulators of GC and affinity-matured Ab responses [59,60]. Secondly, other CD4 T cell subsets might serve different important functions [39], including facilitating optimal CD8 T cell responses. Thirdly, cytotoxic CD8 T cells, which are responsible for the direct killing of pathogen-infected cells by the release of molecules such as granzyme and perforin, are an important “safety net” to generate by vaccination, in case protective Abs fail to completely block a productive viral infection [39]. Additionally, in some instances, vaccine-elicited T cell responses can also correlate with a protective response [37]. Finally, studies in patients with the primary immunodeficiency agammaglobulinemia suggest that in the absence of properly functioning B cell and antibody responses, other cell types (including T cells) might be able to clear SARS-CoV-2 infection with minimal/moderate disease [61,62].

#### 3.2.1. T Follicular Helper Cell Response

Tfh cells, a specialized type of CD4 T cells shaping GC reactions, are of particular interest to vaccine developers because of their crucial importance for the development of GC-derived B cell responses. By delivering co-stimulatory molecules and cytokines to B cells, Tfh cells mediate the formation of GCs and the selection of affinity-matured GC B cells, which can further differentiate into LLPCs or MBCs [59,60]. An effective induction of Tfh cells by the mRNA-LNP platform was previously described in the context of influenza vaccination [31,63]. To verify that this observation could be extended to SARS-CoV-2 mRNA vaccines, we evaluated the induction of Tfh cells after the immunization of BALB/c mice with SARS-CoV-2 mRNA vaccines or with rRBD-AddaVax [24]. As anticipated, the mRNA-LNP platform was a potent inducer of Tfh cells also in the context of a SARS-CoV-2 vaccine, whereas rRBD-AddaVax only induced a more modest Tfh cell population in comparison to SARS-CoV-2 mRNA. The generation of Tfh cells post-SARS-CoV-2 mRNA vaccination was also seen by Tai et al., although the induction was only significant with the RBD-encoding mRNA vaccine, and not with the S1-encoding mRNA [45]. Furthermore, Vogel et al. found a significant increase in the amount of Tfh cells after immunization with BNT162b2 in draining lymph nodes, spleen and blood [12].

Interleukin 21 (IL-21) is a canonical cytokine produced by Tfh cells that can mediate the proliferation of GC B cells and plasma cell differentiation [64]. Of note, our study also revealed that the Tfh cell population driven by SARS-CoV-2 mRNA vaccines was antigen-specific, as measured by the detection of IL-21-producing Tfh cells upon in vitro restimulation with a SARS-CoV-2 peptide pool [24]. In line with our data, Corbett et al. showed that mRNA-1273 was able to promote the generation of IL-21-producing circulating Tfh cells in the blood of NHP-immunized animals, another indicator of robust Tfh cell induction by this vaccine platform [7]. Taken together, these data point at the importance of a vaccine platform that can potently induce Tfh and GC B cells, in a coordinated fashion, with the promise of eliciting long-lasting neutralizing immunity to SARS-CoV-2.

#### 3.2.2. Functional Polarization of CD4 T Cells

The cytokine milieu promoted by a vaccine guides the functional polarization of CD4 T cells. A T helper 1 (Th1)-skewed response is important to vaccine developers because it is the type of immune response required to control intracellular pathogens, such as viruses, as opposed, for instance, to a T helper 2 (Th2) response, which is more critical for the control of helminth infections [39]. In addition, vaccines for respiratory viruses that favor the differentiation of CD4 T cells with a Th2 functional polarization (characterized by the secretion of cytokines such as IL-4 and IL-13) might potentially be detrimental, as they have been linked to vaccine-associated enhanced respiratory disease (VAERD) [48]. In humans, VAERD has only ever been diagnosed in children immunized with inactivated measles and respiratory syncytial virus vaccines, as well as in animal models of respiratory syncytial virus immunization with inactivated virus in alum, a Th2 polarizing adjuvant. The mechanisms involved in VAERD include Th2-biased immune responses. Although no evidence of VAERD has emerged for current SARS-CoV-2 mRNA vaccines, it is important to keep this phenomenon in mind, as the alum-adjuvanted inactivated SARS-CoV vaccine in animals has caused VAERD [48].

In a study by Corbett et al., the authors reported that mice immunized with mRNA-1273 had predominant Th1 response (especially at the highest mRNA vaccine dose), measured by the production of Th1 cytokines IFNγ, TNF, and IL-2 by total CD4 T cells upon in vitro restimulation with SARS-CoV-2 peptide pools [8]. By contrast, mice immunized with the prefusion stabilized recombinant S-2P protein in alum tended to have a Th2-biased response, as indicated by the production of IL-4, IL-5 and IL-13. This finding was confirmed by our group and others, who found that multiple mRNA-LNP vaccine constructs elicited a robust production of Th1 cytokines (including IFNγ, TNF, and IL-2) based on intracellular cytokine staining or ELISpot of total CD4 T cells from lungs and spleen [12,23] or in total splenocytes [42,43]. Similar studies with the vaccines BNT162b2 and mRNA-1273 in rhesus macaques further indicated that total CD4 T cells are heavily Th1-skewed after immunization with both constructs, as indicated by their ability to secrete Th1 but not Th2 cytokines upon stimulation ex vivo [7,12].

Tfh cells are a functionally heterogeneous population. Since different pathogens or vaccine platforms might differentially shape their functional profile, an interesting question is what Tfh cell functional properties are modulated by mRNA-LNP vaccines. In our recently published study [24], we further assessed the quality of the Tfh cell response in SARS-CoV-2 mRNA-immunized mice. To this aim, we measured the polarization of Tfh cells towards either a Th1 or Th2 phenotype, characterized by the production of IFNγ or IL-4, respectively, after in vitro restimulation with a SARS-CoV-2 peptide pool. We demonstrated that mRNA-LNP vaccines skewed Tfh cells towards a Th1 phenotype when using full-length S ∆ furin as immunogen, or towards a mixed Th1/Th2 phenotype when RBD was the immunogen. By contrast, rRBD-AddaVax induced Th2-biased Tfh cells. Of note, the functional polarization of Tfh cells was associated with differential ratios of SARS-CoV-2-binding IgG1 to IgG2a or IgG2b Abs, with rRBD-AddaVax favoring higher IgG1/IgG2a or IgG1/IgG2b ratios than mRNA vaccines [24]. This was an anticipated outcome, as in mice Th2-biased responses are associated with IgG1 production. Conversely, Th1 polarized responses are linked to IgG2 Ab production [65,66,67]. Although Tfh cell responses were not evaluated in this study, similar IgG1 to IgG2a relative ratios were also reported when the IgG responses induced by mRNA-1273 were compared to those driven by S-2P protein formulated in alum [8]. Since a skewing toward vaccine-induced IgG1 production was found in mice developing VAERD in a SARS-CoV model [68], a more balanced IgG1/IgG2a ratio could be a favorable outcome for mRNA vaccines.

Based on these data, it appears that SARS-CoV-2 mRNA-LNP vaccines favor the functional polarization of total CD4 T cells toward Th1, while Tfh cells are characterized by the production of both Th1 (IFNγ) and Th2 (IL-4) cytokines. In keeping with the notion that IL-4 is also an important cytokine produced by Tfh cells, as it mediates an anti-apoptotic helper function on B cells [64], it appears a desirable feature that the mRNA vaccine-induced Tfh cells are capable of retaining some IL-4 production even in a Th1 polarizing cytokine milieu. In line with this idea, in recovered patients with COVID-19, the highest plasma-neutralizing activity was associated with increased frequencies of Th1- and Th2-biased circulating Tfh cells [69]. This finding strengthens the idea that Th1- and Th2-biased Tfh cells are both relevant in shaping a neutralizing response to SARS-CoV-2, and that the simultaneous generation of these two different functional types of Tfh cells could be a favorable feature of SARS-CoV-2 mRNA vaccines.

#### 3.2.3. Cytotoxic T Cell Response

On the whole, mRNA vaccines seem to have a mixed ability to activate CD8 T cell responses. We previously described that a single dose of SARS-CoV-2 mRNA in mice is able to elicit, in both the spleen and the lungs, polyfunctional antigen-specific CD8 T cell responses characterized by the production of IFNγ, IL-2 and/or TNF [23]. Furthermore, the CD8 T cells activated by SARS-CoV-2 mRNA vaccines possessed the typical markers of cytotoxic T cells (Granzyme B and CD107a). In an independent study, Lu et al. found a significant increase in SARS-CoV-2-specific CD8 T cells post-vaccination with SARS-CoV-2 mRNA, measured by IFNγ production on CD8 T cells upon restimulation with vaccine antigens [44].

Preclinical studies of the mRNA clinical candidates also investigated the induction of cytotoxic T cells post-immunization in small and large animal models, with some controversial results. BNT162b2 administration in mice resulted in increased amounts of IFNγ- and IL-2-secreting CD8 T cells in the spleen 12 days after immunization, a result that was mirrored in rhesus macaques [12]. A similar finding was reported in CD8^+^ splenocytes by Rauch et al. with CVnCoV [42]. Additionally, Zhang et al. were able to measure, after vaccination of mice with the clinical candidate ARCoV, a significant expansion of CD8 T effector memory cells, which were likely enriched for SARS-CoV-2-specific cells [43]. On the other hand, Moderna’s mRNA-1273, which elicited a CD8 T cell response in mice [8], failed to induce detectable CD8 T cell responses in preclinical trials in macaques, even with doses as high as 100 μg [7]. A lack of CD8 T cell response to vaccines in which SARS-CoV-2 S is used as the immunogen is not too dissimilar to natural infection in humans, where the S protein has been shown to elicit relatively modest CD8 T cell response only in some, but not all, COVID-19 cases [70]. While potentially beneficial, there is no indication that the induction of cytotoxic CD8 T cells is required for successful protection against SARS-CoV-2 via vaccination.

## 4. Results from Human Clinical Trial with SARS-CoV-2 mRNA Vaccines

The efficacy of a vaccine is often measured by the proportional disease reduction in a population that is vaccinated versus one that has not been vaccinated [71]. Given the strong correlation between vaccine efficacy and the production of protective Abs that has emerged from several decades of human vaccine studies [37], early phase 1/2 research initially relied on this metric to define the efficacy of vaccine candidates. As more data from phase 2 and 3 clinical trials become available, a clear correlation between SARS-CoV-2 mRNA vaccines and protection from COVID-19 is starting to be established.

### 4.1. Phase 1/2

At the time of writing this article, only two vaccine developers, Moderna and BioNTech/Pfizer, have published peer-reviewed phase 1/2 studies on SARS-CoV-2 mRNA vaccines. For this reason, although other mRNA-based vaccine candidates are currently under development, we will focus only on clinical phase 1/2 reports from these two vaccine manufacturers.

#### 4.1.1. Efficacy Profiles: Humoral Responses

In a phase 1 study of mRNA-1273, the vaccine co-developed by the National Institute of Allergy and Infectious Diseases and Moderna, 45 participants received two immunizations (prime and boost) with 25, 100, or 250 μg of this SARS-CoV-2 mRNA vaccine, 28 days apart [6]. Sera from all participants were tested by enzyme-linked immunosorbent assay (ELISA) for full S- and RBD-specific IgG binding titers. Participants in all dosage groups developed anti-SARS-CoV-2 Ab responses, with S-2P- and RBD-specific IgG titers from the 100 and 250 μg vaccine dose groups within the median range of convalescent sera 15 days after initial immunization. Antigen-specific IgG titers continued to increase after the boost, with median post-boost titers from all vaccine dosage groups in the upper range of SARS-CoV-2 convalescent sera controls. Viral neutralization was measured by in vitro neutralization assays with a pseudotyped lentivirus and wild-type SARS-CoV-2. While a dose effect was observed, nAbs reached levels in the range of convalescent serum only after the second immunization in all vaccine groups, indicating the importance of a prime-boost immunization regimen to prompt robust neutralizing responses. Of note, participants from the 100 μg dosage group were followed up to 119 days after the initial vaccination (90 post-boost) and, despite a slight decrease, nAb levels remained significantly elevated in all participants [11]. These data suggest that mRNA-1273 has the potential to induce a durable Ab response. The vaccine candidates BNT162b1 and BNT162b2 were co-developed by BioNTech in collaboration with Pfizer. BNT162b1 encodes a secreted trimerized version of the SARS-CoV-2RBD protein, while BNT162b2 encodes the full-length S protein with the S-2P mutation described earlier. BioNTech and Pfizer concurrently began two phase 1/2 umbrella trials: one with the candidate BNT162b1 in Germany [13] and another one with candidates BNT162b1 and BNT162b2 in the US [14,15]. In the US trial, participants were vaccinated with 10, 30, or 100 μg of BNT162b1 and BNT162b2, while in the German trial doses of 1, 10, 30, 50, or 60 μg of BNT162b1 were tested. All doses of BNT162b1, were able to elicit an RBD-specific IgG response in the range of SARS-CoV-2 convalescent plasma within 21-days of initial vaccination, with an increase in titer after boost [13,14]. Moreover, S-binding IgG Abs were induced by both BNT162b1 and BNT162b2 at relatively comparable levels after the second immunization [15]. Similar to Moderna’s results, both BioNTech’s vaccines elicited nAbs values well above the baseline, measured via an in vitro neutralization assay with a modified SARS-CoV-2 reporter virus, only after the second immunization [13,14,15]. Taken together, these data indicate that SARS-CoV-2 mRNA vaccines are effective at inducing SARS-CoV-2 IgG responses, even at very low dosages. However, a second dose of either mRNA vaccine formulation seems to be required to reach significant levels of nAbs. It is also important to note that only SARS-CoV-2-binding IgG titers and nAb titers were measured in these human trials, and that a deeper analysis of B cell responses post-vaccination will have to be conducted in humans to connect the clinical data with those generated in animal studies.

The elderly population (≥65 years) poses a unique challenge for vaccine developers due to age-associated immunosenescence [72]. Impaired immune responses can potentially reduce vaccine efficacy in elderly subjects and make this population more likely to become critically ill upon infection with SARS-CoV-2. Indeed, the severity of disease and mortality due to COVID-19 are particularly high in the elder population [73]. For these reasons, individuals 65 years of age and older have been studied as a group of special interest. In a small phase 1 dose-escalation trial of mRNA-1273, 40 older adults were stratified according to age (56 to 70 years or ≥71 years) and immunized twice with either 25 or 100 μg of mRNA-1273. The authors found a good reactogenicity profile in both older cohorts, with robust binding and neutralizing Ab responses after two immunizations [9]. The data from this study also suggest that time- and dose-dependent trends were similar to those previously observed in a younger cohort [6], and that individuals over 56 years of age developed higher nAb responses after a second immunization and in response to the higher vaccine dose (100 μg). BioNTech/Pfizer’s candidates were also reviewed in elderly individuals, with both BNT162b1 and BNT162b2 eliciting antigen-specific IgG titers after the first vaccination in elderly individuals that were enhanced by a second immunization [15]. Similar to the younger group, a booster immunization was required in the elderly group to elicit nAbs production, even though nAb titers were overall lower in elder participants in comparison to younger individuals. It is important to note here that since data from both of these companies were from phase 1 trials, the small numbers of participants in each group precluded the authors from performing meaningful statistical analyses.

#### 4.1.2. Efficacy Profiles: Cellular Responses

As discussed earlier, it appears desirable for a SARS-CoV-2 mRNA vaccine to elicit Th1 CD4 T cell responses. Moreover, the induction of SARS-CoV-2-specific cytotoxic CD8 T cells can also be considered favorable, as this could provide an extra layer of immune-mediated protection. In phase 1 trials, Moderna’s vaccine mRNA-1273 displayed measurable SARS-CoV-2-specific total CD4 T cell responses that were strongly biased towards the production of Th1 cytokines, with minimal Th2 cytokine production [6,9]. In both the younger and the elderly study groups, these responses appeared to be higher in individuals receiving 100 μg of mRNA-1273 in comparison to the 25 μg dose group. On the other hand, SARS-CoV-2-specific CD8 T cell responses were almost undetectable in most vaccinated individuals for this vaccine formulation, even after the boost dose. This outcome is not surprising, given that no antigen-specific CD8 T cell responses were found after immunization with mRNA-1273 in rhesus macaques during preclinical trials, even with doses as high as 100 μg [7]. BioNTech/Pfizer’s BNT162b1 vaccine was also able to drive SARS-CoV-2-specific CD4 T cell responses [13]. The SARS-CoV-2-specific total CD4 T cells promoted by BNT162b1 were polarized toward a Th1 functional profile, as measured by the frequency of SARS-CoV-2-specific CD4 T cells producing IFNγ and IL-2 but not IL-4, upon stimulation with SARS-CoV-2 peptides. Differently from mRNA-1273, BNT162b1 was able to drive a SARS-CoV-2-specific CD8 T cell response in the majority of the trial subjects [13]. The phase 1/2 data are in line with all the animal studies on different mRNA vaccines described in 3.2.3, thus confirming that mRNA-1273 seems to be unique in its inability to trigger SARS-CoV-2-specific CD8 T cell response, while other mRNA vaccines are capable of inducing detectable virus-specific CD8 T cells. It is unclear why mRNA-1273 is unable to promote effective CD8 T cell response in larger animal models and humans. Possible contributors might be the SARS-CoV-2 mRNA design or the formulation of the LNPs.

#### 4.1.3. Safety Profiles

These mRNA vaccine candidates have relatively favorable safety profiles. mRNA-1273 had no serious adverse events reported during the phase 1 trial that met the criteria for halting the trial. Among the reported adverse events, local pain at the injection site was common, whereas systemic events including fever, chills and headache were registered with increased incidence and severity after the booster immunization and with the higher vaccine doses [6]. The same safety profile was also observed in the elderly population [9].

The safety profiles of BNT162b1 and BNT162b2 are very similar to that of mRNA-1273 [13,14,15]. All vaccine groups reported pain and tenderness at the injection site as the most common adverse event. Fever, fatigue and chills were the most frequently reported systemic adverse events. Reactogenicity was dose-dependent and was more pronounced after the boost dose. On the basis of the higher reactogenicity reported at higher doses upon one immunization and after the boost at the lower vaccine doses, participants who received an initial 100 μg [14] or 60 μg [13] dose did not receive a second vaccine injection. Importantly, BNT162b2 induced less adverse events, including fever and chills, than BNT162b1, particularly in participants aged 65–85 years. The lower reactogenicity of BNT162b2, along with a comparable immunogenicity of the two candidates, supported the advancement of BNT162b2 to subsequent phase 3 studies [15].

### 4.2. Phase 3 Trials

As of 30 December 2020, the mRNA vaccine candidates BNT162b2 from BioNTech/Pfizer and mRNA-1273 from Moderna have both been approved for emergency use authorization in the United States. As such, mRNA vaccine administration to the public has commenced. Although necessary to halt the SARS-CoV-2 pandemic, with this decision also comes the issue of confounding the continuation of phase 3 studies. The individuals originally in the placebo control groups will also be vaccinated, making the long-term double-blinded study of SARS-CoV-2 vaccine-induced immunity impossible.

BioNTech/Pfizer have recently published data from the ongoing phase 2/3 clinical trial that is testing their vaccine, BNT162b2, for both safety and efficacy [16]. About 44,000 participants are enrolled in the trial, with ages ranging from 16 to 85 years of age. The participants were split randomly, at a 1:1 ratio, into two groups to receive either BNT162b2 or a placebo. The injections were given as a two-dose series, whereby the BNT162b2 group received two 30 μg doses, 21 days apart, and the placebo group received two saline injections instead. This two-dose immunization regimen conferred a remarkable 95% protection against COVID-19, citing that of those who received BNT162b2, only eight developed COVID-19, whereas 162 cases were reported in the placebo group. BNT162b2 displayed a very favorable safety profile, with only 27% of the BNT162b2 group and 12% of the placebo group reporting adverse events. These events were mostly short-term mild-to-moderate local reactions, such as pain at the injection site, and short-term systemic reactions such as fatigue, headache, and fever.

The Moderna vaccine mRNA-1273 is also currently undergoing a phase 3 clinical trial, data from which has recently been published [10]. This trial consists of around 30,000 participants with ages ranging from 18 to 85 years of age who were split randomly, at a 1:1 ratio, into two groups to receive either mRNA-1273 or a placebo. All participants received a two-dose injection series of either 100 μg of mRNA-1273 or a saline placebo, separated by 28 days. Moderna reported a 94.1% efficacy rate for mRNA-1273. Only 11 COVID-19 cases were reported in the mRNA-1273 vaccine group, versus 185 cases in the placebo group. The safety profile of this vaccine was also very favorable, with no specific safety concerns identified.

While both BNT162b2 and mRNA-1273 are currently approved for emergency use authorization, it is important to remember that the phase 3 trials are not complete. Participants will continue to be monitored over the coming months, and potentially years, to fully assess the vaccine safety and efficacy.

## 5. Strengths and Limitations of SARS-CoV-2 mRNA Vaccine Candidates

### 5.1. Strengths

mRNA-based vaccines have become an increasingly attractive platform to fight the ongoing SARS-CoV-2 pandemic for a multitude of reasons. Firstly, the need for only a DNA template of the desired antigen to produce a vaccine candidate, resulting in an exceedingly fast manufacturing timeline [4]. Clinical testing of the first mRNA vaccine candidate (mRNA-1273) began on March 2020 [74], just 66 days after the SARS-CoV-2 sequence was publicly released on January 2020 [75], with the second candidates (BNT162b1 and BN162b2) entering into phase 1/2 clinical trials only a month later [76,77]. By contrast, the development of vaccine candidates utilizing traditional vaccine platforms has been lengthy because of the inherently slow nature of developing cell lines, generating virus, and/or producing clinical-grade protein subunits, as demonstrated by the fact that the only vaccines that have been granted emergency authorization by the FDA thus far are mRNA vaccines.

Secondly, mRNA vaccines elicited a very potent immune response in both animal studies and human clinical trials, as extensively discussed in Section 3 and Section 4. Importantly, these potent immune responses are substantiated by an impressive protection from COVID-19 in phase 2/3 studies [10,16]. While the FDA has initially stated that SARS-CoV-2 vaccines will require a minimum of 50% efficacy to qualify for approval [78], both of the current mRNA vaccines currently approved for emergency use authorization (mRNA-1273 and BNT162b2) reported a greater than 94% efficacy [10,16]. This level of efficacy sets a very high standard for SARS-CoV-2 vaccines, considering that the next most-advanced vaccine candidate in clinical trials (ChAdOx1 nCoV-19), an adenoviral vector vaccine from AstraZeneca/University of Oxford, is only about 70% effective [79]. 

mRNA vaccines also possess additional desirable features. Compared to other vaccine platforms, mRNA vaccines are appealing because of their minimalist nature. mRNA vaccines do not need a vector for their delivery/expression, thus removing the possible complication of pre-existing and/or de novo anti-vector immunity [80]. Differently from inactivated or attenuated vaccines, less important antigenic targets that do not lead to nAb generation are not included. Since there is no need for the involvement of any viral growth, the possibility of other contaminating viruses from the cell lines is removed. mRNA vaccines that are encapsulated in LNPs also do not require complex delivery methods involving electroporation, as required by DNA vaccines, nor do they need the addition of an adjuvant, which is required with protein vaccines. Moreover, as described earlier, all available data suggest that the mRNA-LNP platform polarizes T cells towards a Th1 bias, suggesting that the likelihood of these vaccines causing adverse events, such as VAERD (discussed in Section 3.2.2), seems quite remote.

Finally, mRNA vaccines can be readily modified based on need. Target immunogenic epitopes can be easily switched in and out of candidates, as all that is needed is the DNA sequence of the antigen to serve as a template. A SARS-CoV-2 vaccine construct can be quickly adjusted to target a newly emerged coronavirus strain [8].

### 5.2. Challenges and Limitations

Although all the clinical data generated thus far indicate that mRNA vaccines are safe to use in humans, this is the first time that a vaccine of this type has been licensed. With that comes some potential unknowns. For example, there have been rare, recent reports of individuals experiencing anaphylaxis following immunization with a COVID-19 mRNA vaccine [81]. As with all vaccines, some people can have allergic reactions to one or more of the components that make up the vaccine. As it stands, it is unclear what the offending component(s) of this mRNA vaccine is (are), but anaphylaxis represents a major concern for people with a history of severe allergies. 

Another uncertainty is the length of the immunity conferred by SARS-CoV-2 mRNA vaccines in humans. All preclinical data indicate that there might be long-term immunity elicited by this platform in animal models, but extended studies to address this point have not been published yet. In experiments utilizing an influenza hemagglutinin (HA)-based mRNA-LNP vaccine, it was shown that HA inhibition titers only slightly decrease in mice up to 400 days post-immunization [31], indicating that this vaccine platform is capable of generating durable Ab responses. To date, the persistence of mRNA vaccine-induced Abs in humans has only been reported up to 119 days post-initial immunization [11]. These data do, however, indicate that SARS-CoV-2-specific Ab titers only slightly wane in this time period, indicating the potential for long-lasting vaccine-induced immunity with this platform.

The need for ultracold storage required by mRNA vaccines is also a concern. Some research has suggested, however, that these vaccines are fairly stable at 4 °C for a week [43]. As it stands, the current recommendations for storage of the mRNA vaccine BNT162b2 are −80 °C to −60 °C for up to 6 months, or 2 °C to 8 °C for up to 5 days, to preserve potency [82]. Recommendations for the storage of the Moderna vaccine, mRNA-1273, are slightly different, with the FDA recommending long-term storage at −25 °C to −15 °C and storage at 2 °C to 8 °C for up to 30 days [83]. While the transportation and storage in many countries will not be an insurmountable issue, a wide distribution in remote locations and in developing countries appears quite challenging for mRNA vaccines.

## 6. Conclusions

Moving from the release of the SARS-CoV-2 genome sequence to SARS-CoV-2 mRNA vaccine phase 1/2 clinical trials within a couple of months has been an unprecedented endeavor for vaccine developers. Usually, the development of vaccines using standard approaches requires multiple years before an effective vaccine is licensed for human use. With the expedited development of SARS-CoV-2 mRNA vaccines comes public concern over cutting corners, and a fear of releasing a vaccine to the public before safety and efficacy are deeply assessed. Herein, we have provided a synopsis of SARS-CoV-2 mRNA vaccines, with emphasis on the adaptive immune responses elicited by the vaccine in preclinical and clinical studies. Additionally, we have laid out the pros and cons of the mRNA vaccine platform. Published data from the current clinical candidates demonstrate a high degree of both efficacy and safety for this vaccine modality. Based on the available data, the mRNA vaccine platform offers a paradigm shift for both the development of vaccines as well as the preparedness for future pandemics.

## Supplementary Materials

The following are available online at https://www.mdpi.com/2076-393X/9/2/147/s1.

## Abbreviations

Ab	Antibody
ACE2	Angiotensin-converting enzyme 2
APC	Antigen-presenting cell
COVID-19	Coronavirus Disease 2019
Δfurin	Full-length S lacking the furin cleavage site
DC ELISA	Dendritic cell Enzyme-linked immunosorbent assay
GC HA	Germinal center Hemagglutinin
Ig	Immunoglobulin
IL	Interleukin
LLPC	Long-lived plasma cell
LNP	Lipid nanoparticle
m1Ψ	N1-methyl-pseudouridine
MBC	Memory B cell
mRNA	Messenger RNA
nAb	Neutralizing antibody
RBD	Receptor binding domain (of SARS-CoV-2 S)
RIG-I	Retinoic acid inducible gene I
rRBD	Recombinant RBD
rRBD-AddaVax	rRBD given with the adjuvant AddaVax
S	Spike protein (of SARS-CoV-2)
S-2P	Spike 2-proline (SARS-CoV-2 S with proline substitutions at amino acids 986 and 987; stabilized in a prefusion conformation)
S1	Subunit 1 (of SARS-CoV-2 S)
S2	Subunit 2 (of SARS-CoV-2 S)
SARS-CoV-2	Severe Acute Respiratory Syndrome Coronavirus 2
Tfh	T follicular helper cells
Th1	T helper 1
Th2	T helper 2
TLR	Toll-like receptor
