# Supplementary material for: SARS-CoV-2 mRNA Vaccines: Immunological Mechanism and Beyond
Source: Vaccines (Basel). 2021 Feb 12;9(2):147. doi: 10.3390/vaccines9020147 (PMC7918810; doi:10.3390/vaccines9020147)
Supplement: Supplementary file 1 [file vaccines-09-00147-s001.pdf]

mRNA Vaccine Constructs	Translated Protein	Construct Used By
**Full-length S**<br>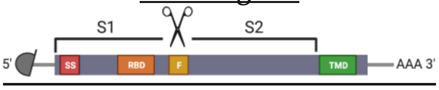 	- Full-length spike protein.<br>- Expressed on the cell surface.<br>- Will be cleaved.	- Lu *et al.* [44]
**Full-length S-2P**<br>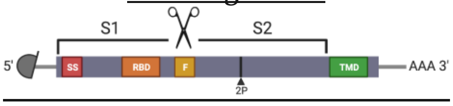 	- Full-length spike protein with 2P mutations at a.a. 986 and 987.<br>- Expressed on cell surface.<br>- Will be cleaved but is prefusion stabilized.	- mRNA-1273 [6–11]<br>- BNT162b2 [12,15,16]<br>- CVnCoV [42,47]
**Full-length S-Δfurin**<br>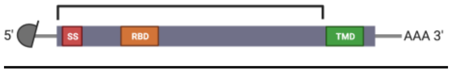 	- Full-length spike protein with furin deletion.<br>- Expressed on cell surface.<br>- Will not be cleaved.	- Laczkó *et al.* [23]<br>- Lederer *et al.* [24]
**S1**<br>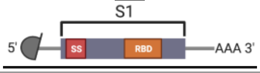 	- S1 only.<br>- Secreted from cell.	- Tai *et al.* [45]
**RBD**<br>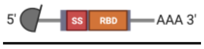 	- RBD only.<br>- Secreted from cell.	- BNT162b1 [13–15]<br>- Laczkó *et al.* [23]<br>- Lederer *et al.* [24]<br>- ARCoV [43]<br>- Lu *et al.* [44]<br>- Tai *et al.* [45]

Figure S1. SARS-CoV-2 mRNA vaccine constructs. (1) The full-length mRNA construct, which encodes the wild-type spike protein, is only used by one study covered in this review. Due to changes in the accessibility of conformational epitopes after cleavage and fusion, which may result in suboptimal antibody responses, this construct is not often used in vaccine design. (2) The full-length S-2P mRNA construct is used in most current clinical vaccine formulations. The 2P mutation allows for cleavage of the spike protein while still leaving prefusion conformational epitopes accessible. (3) The full-length Δfurin construct is an uncleavable version of the spike protein which also preserves conformational epitopes. (4) The S1-only construct was used by Tai *et al.*, and contains only the S1 portion of the spike protein, including the RBD. (5) The RBD-only construct is used by numerous groups, as it contains many epitopes important for SARS-CoV-2 neutralization. SS: signal sequence; RBD: receptor binding domain; F: furin cleavage site; TMD: trans-membrane domain.
